# Blood pressure patterns and body mass index status in pregnancy: An assessment among women reporting for antenatal care at the Korle-Bu Teaching hospital, Ghana

**DOI:** 10.1371/journal.pone.0188671

**Published:** 2017-12-06

**Authors:** Mary Amoakoh-Coleman, Deda Ogum-Alangea, Emefa Modey-Amoah, Michael Yao Ntumy, Richard M. Adanu, Samuel A. Oppong

**Affiliations:** 1 Department of Epidemiology, Noguchi Memorial Institute for Medical Research, University of Ghana, Legon, Ghana; 2 Department of Epidemiology and Disease Control, School of Public Health, University of Ghana, Legon, Ghana; 3 Julius Global Health, Julius Center for Health Sciences and Primary Care, University Medical Centre, Utrecht, The Netherlands; 4 Department of Population, Family and Reproductive Health, School of Public Health, University of Ghana, Legon, Ghana; 5 Department of Obstetrics and Gynecology, School of Medicine and Dentistry, University of Ghana, Accra, Ghana; Centro Cardiologico Monzino, ITALY

## Abstract

**Background:**

Maternal obesity in pregnancy has been linked with increased risk of pregnancy induced hypertension (PIH). In some tertiary referral hospitals in Ghana, PIH is the leading cause of institutional maternal mortality.

**Objective:**

To evaluate blood pressure changes during pregnancy amongst different body mass index (BMI) groups and how this relates to the risk of developing PIH.

**Methods:**

Women who had a dating ultrasound before 20 weeks gestation and registering for antenatal care at the Korle-Bu Teaching Hospital in Accra, between February and December 2013 and met the inclusion criteria were recruited into a cohort study. BMI was assessed at baseline. Blood pressure measurements were taken at (±2) 24, 28 and 36 weeks. Primary outcome measure of interest during follow-up was a diagnosis of PIH at these points. BP changes during follow up at the three points were measured. Descriptive analysis of baseline factors was carried out and compared for the BMI groups. Relative risk (RR) of PIH was estimated at 95% confidence interval.

**Results:**

Mean (SD) age for the 361 women was 30.9 (4.8) years. Incidence of PIH amongst the cohort was 10.5% (95% CI: 7.45% - 14.45%) and 40.4% and 33.0% of them were overweight and obese respectively at baseline. Pregnant women who were obese at baseline had a three-fold increased risk of PIH compared to those with normal BMI [RR = 3.01 (1.06–8.52), p = 0.04].

**Conclusion:**

Obese women have a significantly increased risk of PIH. Women should be screened at booking for obesity status. Antenatal protocols should have interventions for prevention or early detection of obesity and management of obesity to improve outcomes.

## Introduction

The global epidemic of non-communicable disease (NCDs) has been linked to obesity, with a highest prevalence in high income countries[1–3]. Obesity has been shown not only to be prevalent in high-income countries but also display rising prevalence in low and middle-income countries (LMIC)[4]. A survey of seven Sub Saharan African countries shows that obesity prevalence increased by an average of 35% in the last two decades with some estimates reaching as high as 50% [5]. Other studies estimate a doubling of obesity prevalence in the last 15 years, in West Africa, especially amongst women[4]

Studies in Ghana indicate that obesity prevalence among Ghanaian women ranges between 7.4–50%[6–11]. The obesity epidemic in Ghana is most notable among women in the reproductive age group. Data from the 2008 Ghana Demographic and Health Survey (GDHS) suggests that 29% of pregnant women are overweight and 12% are obese[11]. Two hospital-based, surveys conducted in Ghana and Nigeria reported the prevalence of obesity in pregnancy to be 17.9% and 6.0% respectively[12,13].

Maternal obesity and excessive weight gain in pregnancy have been linked with increased risk of pregnancy induced hypertension and consequently maternal and perinatal morbidity and mortality[13–15]. Globally, hypertensive disease in pregnancy is the second direct cause of maternal mortality[16]. It contributes 19–40% of maternal morbidity and deaths in Ghana, second only to hemorrhage[17,18]. Recent reports from some tertiary referral hospitals in Ghana indicate that hypertensive disease in pregnancy is now the leading cause of institutional maternal mortality[19,20].

To date, interventions to control obesity and excessive weight gain during pregnancy have not been implemented in Ghana. Although guidelines and protocols recognize hypertension and diabetes, among others as independent risk factors for adverse outcome during pregnancy, they are limited in their focus on the effect of obesity during pregnancy. Risk of obesity associated PIH has not been determined. Our aim was to evaluate blood pressure changes during pregnancy amongst different body mass index (BMI) groups and how this relates to the risk of developing PIH.

## Materials and methods

A cohort study of pregnant women registering for antenatal care at the Korle-Bu Teaching hospital, in Accra was conducted.

Accra was chosen because of its relatively high prevalence of obesity among women of reproductive age and high antenatal coverage and skilled birth attendants rates[21,22]. The Korle-Bu Teaching hospital is a referral hospital and registers about 1000 new antenatal attendants every month with about 11,000 deliveries each year (figure from 2011 hospital report); which makes it an ideal site for this study. At each antenatal visit, weight, height and blood pressure measurements are taken amongst others. Urine dipstick test for glucose and protein are routinely done for all clients.

The sample size was computed using a risk ratio of 14% vs. 6% for hypertension in pregnancy, in the obese and the non-obese (underweight, normal and overweight) group respectively. To detect a statistical difference, at a power of 80% and a 95% confidence interval, a sample of 230 women was required for each group. An attrition rate of 20% was assumed based on estimates of 15–20% for longitudinal studies. Total sample size required in each group was 300 women, totaling 600.

All pregnant women 18 years and above attending antenatal care at the Korle-Bu Teaching Hospital between the three months recruitment period with singleton pregnancy at 20 to 24 weeks gestation were identified and screened to ensure they meet the study criteria. Women sampled were categorized into four groups according to their body mass index.

Inclusion Criteria: Women who had a dating ultrasound before 20 weeks gestation. Women were excluded from the study if they registered after 24 weeks, had multiple pregnancy, had booking blood pressure of 140/90 mmHg or higher or on antihypertensive medication or was a known diabetic. Clients with chronic medical illness such as sickle cell disease, HIV, severe anaemia, or severely underweight or any deformity that interfered with weight and height measurements, were also excluded.

All first time antenatal clients were approached and screened with our screening tool. An approved written consent form was administered to those who qualified based on our inclusion and exclusion criteria. The women were weighed wearing only light clothing using recalibrated weighing scale (HealthCare). Blood pressure was measured after clients have been made to rest for 30 minutes and measurement taken from the right arm in the sitting position using a standard adult size arm cuff sphygmomanometer (M2 Basic, OMRON Intelli^TM^ sense). Participant’s height was measured using a stadiometer without wearing any footwear ((HealthCare). Weight and height were measured twice and the average recorded. Pregnancy was dated using combination of last menstrual period (LMP) and ultrasound performed before twenty weeks. Where the LMP cannot be reliably recalled, a first trimester dating ultrasound was used. A structured questionnaire was used to collect baseline data on socio-demographic information, relevant medical history, past and current obstetric history, contraceptive and drug history. The gestational age at booking, maternal weight, height and blood pressure measurements at booking visit were also recorded. Folders of the recruited participants were tagged to indicate participation in the study. Folder tags included a data entry sheet for records such as delivery information and post-delivery outcomes (i.e. gestational age at delivery; type of delivery; birth weight; maternal complications like eclampsia, postpartum hemorrhage; presentation of baby at birth and NICU admission).

All participants were managed by the usual antenatal care protocol at the Korle-Bu Teaching hospital. In addition all participants had BP measurements taken at (+/-2) 24, 28 and 36 weeks as described above. Other measures of antenatal care were taken and at delivery a final set of outcome data were collected.

Primary outcome measure of interest during follow-up was a diagnosis of gestational hypertension or pregnancy induced hypertension (PIH) at or around (+/- 2) 24, 28 and 36 weeks of gestation. Secondary outcome measures of interest during follow-up were mean systolic and diastolic BP changes during follow up at or around (+/- 2) weeks 24, 28 and 36 weeks.

The diagnosis of gestational hypertension for clients in the study was defined as any de novo record of systolic BP of at least 140 mmHg and/or a diastolic BP of at least 90 mmHg after 20 weeks of gestation. Measurements were taken at 24, 28 and 36 weeks of gestation.

Data was entered into SPSS version 16. Descriptive analysis of women at baseline was carried out and compared for the BMI groups. Continuous variables such as weight, height and blood pressure measurements were compared using the student’s t-test while Chi-Square tests were used to determine associations between categorical variables.

Mean BP changes for the BMI groups were depicted in graphs. Linear regression was used to assess relationships between the outcome and maternal BMI at first visit and BP changes during the pregnancy at 24, 28 and 36 weeks of gestation. Risk for PIH amongst different BMI categories was assessed using logistic regression. Statistical significance was determined at p<0.05 at the 95% confidence level.

### Ethical approval

Ethical approval was obtained from the Ethical Review Board of the Noguchi Memorial Institute for Medical Research, University of Ghana, Legon (Protocol number NMIMR-IRB 042/12-13) before data collection commenced. Permission was also obtained from the Korle-bu Teaching Hospital before any contact was made with participants. A written informed consent was obtained from each participant recruited.

## Results

### Baseline characteristics

**Fig 1:**
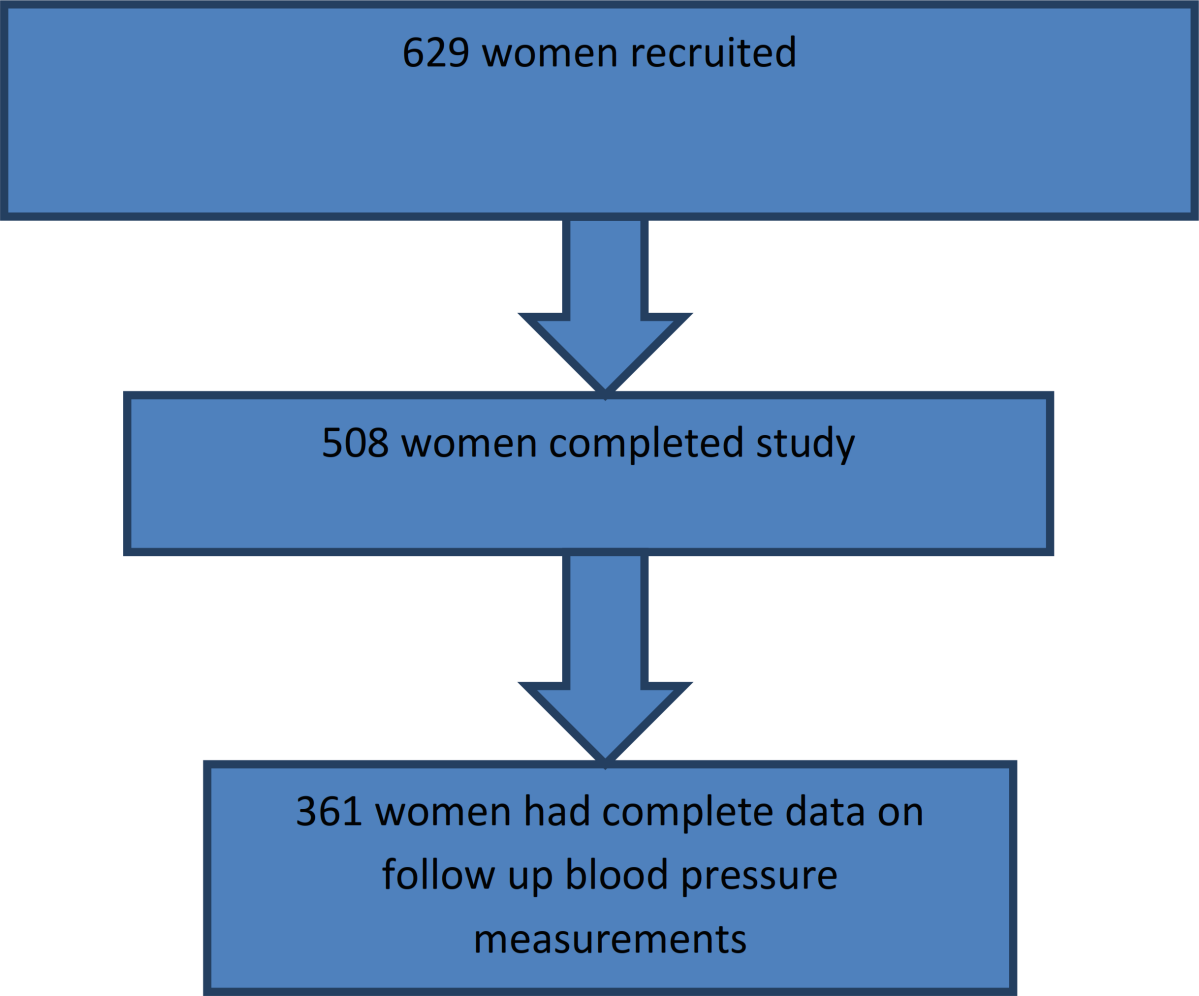
Flow chart of participants in the study.

A total of 629 women were recruited from March 2013 –December 2013 but only 508 women completed the study. This gave us a loss to follow-up rate of 19.2%. For the 508 who completed the study, the outcome measure of PIH diagnosis was obtained for 361 women, translating to missing data of 28.9%. We did a complete case analysis for the 361 women, as the baseline characteristics, including BMI status, of those without data on the outcome were similar to that of those with data. Also because data on outcome for the obese was less compared to the non-obese groups, we decided to do analysis for each of the BMI groups instead of the originally intended obese and non-obese categorization. "Fig 1" is the representation of participants flow through the study.

The mean (SD) age of the women was 30.9 (4.8) years, weight was 73.2 (14.5) kg; height of 160.6 (5.8) cm and gestational age of 20.5 (1.9) weeks with hemoglobin of 11.1 (1.2) g/dl at baseline. A total of 65.4% of them had secondary or higher education with 2.8% having no education. Most of them were employed (90.9%), married (90.0%) and Christians (87.8%). Also at baseline, 25.8% had normal BMI, 40.4% were overweight and 33.0% were obese. Underweight women were only 3 (0.8%). The overweight and obese women were significantly older, multiparous, Christian, and had higher baseline SBP and DBP. Variations in BMI were mainly due to differences in weight, with mean weight (SD) of normal, overweight and obese being 58.8 (6.2) kg, 69.8 (6.4) kg and 88.8 (11.6) kg respectively (p<0.01). They however did not differ from the normal BMI group with respect to education, employment and residence. The mean (SD) systolic BP and diastolic BPs for participants were 111.0 (11.1) mmHg and 68.9 (9.3) mmHg respectively. A total of 22 (6.10%) of participants had systolic blood pressure of 130 mmHg or more, with majority of these being obese (63.60%). The baseline BPs of the groups was significantly different. The mean systolic BP of the obese was 113.00 mmHg compared to that of the normal BMI group of 108.2 mmHg (p = 0.03) while mean diastolic BP of the obese was 71.2 mmHg compared to 66.5 mmHg for the normal BMI women (p<0.01). More obese (11.8%) and overweight (4.8) women had higher systolic BPs compared to normal BMI women (p = 0.01) at baseline. Table 1 describes the baseline characteristics of the women.

**Table 1:** Baseline characteristics of participants and comparison among different BMI groups.

Variable	All Participants Frequency N (%) / Mean (SD)	Underweight	Normal	Overweight	Obese	p-value
BMI						
Underweight Normal Overweight Obese	3 (0.8)					
	93 (25.8)					
	146 (40.4)					
	119 (33.0)					
Education						
None Primary Secondary Tertiary	10 (2.8)	0 (0.0)	2 (3.6)	4 (2.7)	4 (3.4)	
	115 (31.9)	1 (33.3)	23 (41.8)	52 (35.6)	39 (32.8)	
	91 (25.2)	2 (66.7)	30 (54.5)	38 (26.0)	21 (17.6)	
	145 (40.2)	0 (0.0)	0 (0.0)	52 (35.6)	55 (46.2)	
Employed						
No Yes	32 (8.9)	0 (0.0)	11 (11.8)	14 (9.6)	7 (5.9)	
	328 (90.9)	3 (100.0)	82 (88.2)	132 (90.4)	112 (94.1)	
Religion						
Christian Muslim Other	317 (87.8)	1 (33.3)	79 (84.9)	128 (88.9)	109 (92.4)	
	41 (11.4)	2 (66.7)	14 (15.1)	16 (11.1)	9 (7.6)	
	3 (0.8)					
Marital status						
Single Married Cohabiting	22 (6.1)	0 (0.0)	6 (6.5)	10 (6.8)	6 (5.0)	
	325 (90.0)	3 (100.0)	84 (90.3)	130 (89.0)	108 (90.8)	
	3 (0.8)	0 (0.0)	3 (3.2)	6 (4.1)	5 (4.2)	
Ethnicity						
Akan Ga/ Dangme Ewe Northern	211 (58.4)	3 (100.0)	57 (62.0)	86 (58.9)	65 (55.1)	
	84 (23.3)	0 (0.0)	15 (16.3)	23 (15.8)	14 (11.9)	
	52 (14.4)	0 (0.0)	16 (17.4)	32 (21.9)	36 (30.5)	
	12 (3.3)	0 (0.0)	4 (4.3)	5 (3.4)	3 (2.5)	0.63
Residence						
Rural Urban	11 (3.0)	0 (0.0)	4 (4.3)	2 (1.4)	5 (4.2)	
	349 (96.8)	3 (100.0)	88 (95.7)	144 (98.7)	113 (95.8)	
Age (years)	30.90 (4.81)	27.67 (2.52)	29.56 (5.05)	30.80 (4.70)	32.16 (4.49)	<0.01
Parity	1.11 (1.10)	1.00 (1.00)	0.68 (0.93)	1.10 (1.12)	1.46 (1.14)	<0.01
Weight (kg)	73.16 (14.54)	46.67 (6.66)	58.79 (6.19)	69.78 (6.38)	88.84 (11.62)	<0.01
Height (cm)	160.60 (5.78)	161.33 (8.500	160.87 (5.93)	159.81 (5.58)	160.65 (5.83)	0.15
Gestational age at booking (wks)	20.46 (1.85)	22.67 (1.16)	20.47 (1.84)	20.52 (1.76)	20.46 (1.92)	0.17
Baseline systolic BP (mmHg)	110.99 (11.13)	109.00 (11.35)	108.19 (10.35)	111.49 (10.54)	112.99 (12.09)	0.03
Baseline diastolic BP (mmHg)	68.87 (9.31)	59.33 (2.08)	66.47 (9.49)	68.73 (8.42)	71.16 (9.75)	<0.01
Baseline systolic BP > 130mmHg	22 (6.10)	0 (0.00)	1 (4.50)	7 (31.80)	14 (63.60)	0.01
Hemoglobin (g/dl)	11.07 (1.22)	11.03 (0.25)	11.11 (1.13)	11.1 (1.13)	11.00 (1.29)	0.94

### Risk of PIH and associated factors

Overall, the incidence of PIH amongst our cohort was 10.5% (95% CI: 7.45% - 14.45%). Majority of these were overweight (40.4%) and obese (33.0%) at baseline. Risks of systolic and diastolic PIH at 36 weeks were 4.2% and 2.8% respectively. Risks of PIH (either systolic or diastolic) at 24, 28 and 36 weeks were 1.7%, 5.3% and 5.8% respectively. Risk of pre-eclampsia and eclampsia was 3.9%. There were no significant differences between the groups that were diagnosed with PIH and those who were not with respect to age, parity, height and gestational age at booking. There were no significant differences between the two groups at baseline with respect to socioeconomic characteristics. There were more obese women at baseline who developed PIH (44.7%) compared to overweight women (34.2%). Consequently, there were fewer obese women (31.6%) than overweight women (41.2) at baseline who did not develop PIH during follow-up. Table 2 describes the characteristics of the women with and without PIH.

**Table 2:** Association of baseline characteristics with diagnosis of PIH among cohort.

Variable		PIH No	PIH Yes	p-value
	Incidence	323 (89.5)	38 (10.5)	
BMI				
	Underweight	2 (0.6)	1 (33.3)	0.19
	Normal	86 (26.6)	7 (18.4)	
	Overweight	133 (41.2)	13 (34.2)	
	Obese	102 (31.6)	17 (44.7)	
Education				0.34
	None	10 (3.1)	0 (0.0)	
	Primary	99 (30.7)	16 (42.1)	
	Secondary	81 (25.1)	10 (26.3)	
	Tertiary	133 (41.2)	12 (31.6)	
Employed				0.33
	No	27 (8.4)	5 (13.2)	
	Yes	295 (91.6)	33 (86.8)	
Religion				0.73
	Christian	284 (88.2)	33 (86.8)	
	Muslim	36 (11.8)	5 (13.2)	
Marital status				0.11
	Single	22 (6.8)	0 (0.0)	
	Married	290 (89.5)	35 (92.1)	
	Cohabiting	11 (3.4)	3 (7.9)	
Ethnicity				0.87
	Akan	189 (58.7)	22 (57.9)	
	Ga/ Dangme	77 (23.9)	7 (18.4)	
	Ewe	46 (14.3)	6 (15.8)	
	Northern	10 (3.1)	2 (5.3)	
Residence				0.95
	Rural	10 (3.1)	1 (2.6)	
	Urban	312 (96.9)	37 (97.4)	
Age (years)		30.87 (4.78)	31.16 (5.16)	0.73
Parity		1.11 (1.09)	1.05 (1.18)	0.74
Weight (kg)		72.66 (14.29)	77.39 (10.08)	0.05
Height (cm)		160.56 (5.75)	160.97 (5.91)	0.68
Gestational age at booking (wks)		20.49 (1.70)	20.18 (2.85)	0.34
Baseline systolic BP (mmHg)		109.97 (10.62)	119.68 (11.66)	<0.01
Baseline diastolic BP (mmHg)		67.95 (8.63)	76.76 (11.18)	<0.01
Baseline systolic BP > 130 (mmHg)	Yes	13 (4.00)	9 (23. 70)	<0.01
Hemoglobin (g/dl)		11.01 (1.21)	11.59 (11.18)	0.02

There was significant difference in mean baseline hemoglobin of women diagnosed with PIH compared to those without PIH (11.6g/dl vs 11.0g/dl, p = 0.02). Women diagnosed with PIH recorded significantly higher mean systolic and diastolic BP at baseline compared to their non-PIH counterparts (119.7/76.8 mmHg vs 110.0/68.0 mmHg, p<0.01).

There is a significantly higher risk of PIH in obese women compared to women with normal BMI [RR 3.01 (1.06–8.52), p = 0.04]. However, the elevated risk of PIH among overweight compared to women of normal weight was not statistically significant [RR 1.54 (0.55–4.35), p = 0.41]. Although only 9 (40.90%) of those with baseline systolic BP higher than 130 mmHg developed PIH, the crude relative risk of developing PIH if baseline systolic BP is higher than 130 mmHg was crude RR 7.40 [(2.92–18.78), p<0.001]. When adjusted for socioeconomic factors as well as BMI category, baseline systolic BP higher than 130mmHg was still associated with increased risk of developing PIH over six-fold [adjusted RR 6.59 (2.33–18.67), p<0.001), Table 3.

**Table 3:** Risk of PIH among the BMI groups.

Body Mass Index category	RR	95% CI	p-value
Crude			
Underweight	6.14	0.49–76.43	0.16
Normal	-	-	-
Overweight	1.20	0.46–3.13	0.71
Obese	2.05	0.81–5.17	0.13
Adjusted for baseline characteristics*			
Underweight	20.27	0.98–418.41	0.05
Normal	-	-	-
Overweight	1.54	0.55–4.35	0.41
Obese	3.01	1.06–8.52	0.04
Adjusted for baseline characteristics and GDM at 28 weeks			
Underweight	6.28	0.29–137.10	0.24
Normal	-	-	-
Overweight	1.28	0.41–3.96	0.67
Obese	2.72	0.85–8.76	0.09
Risk of PIH amongst those with baseline systolic BP higher than 130 mmHg**			
	6.59	2.33–18.67	<0.001

* These are age, parity, religion, ethnicity, gestational age at booking, and baseline systolic and diastolic blood pressures

** Adjusted for baseline factors and baseline BMI category

### Blood pressure changes during follow up

**Fig 2:**
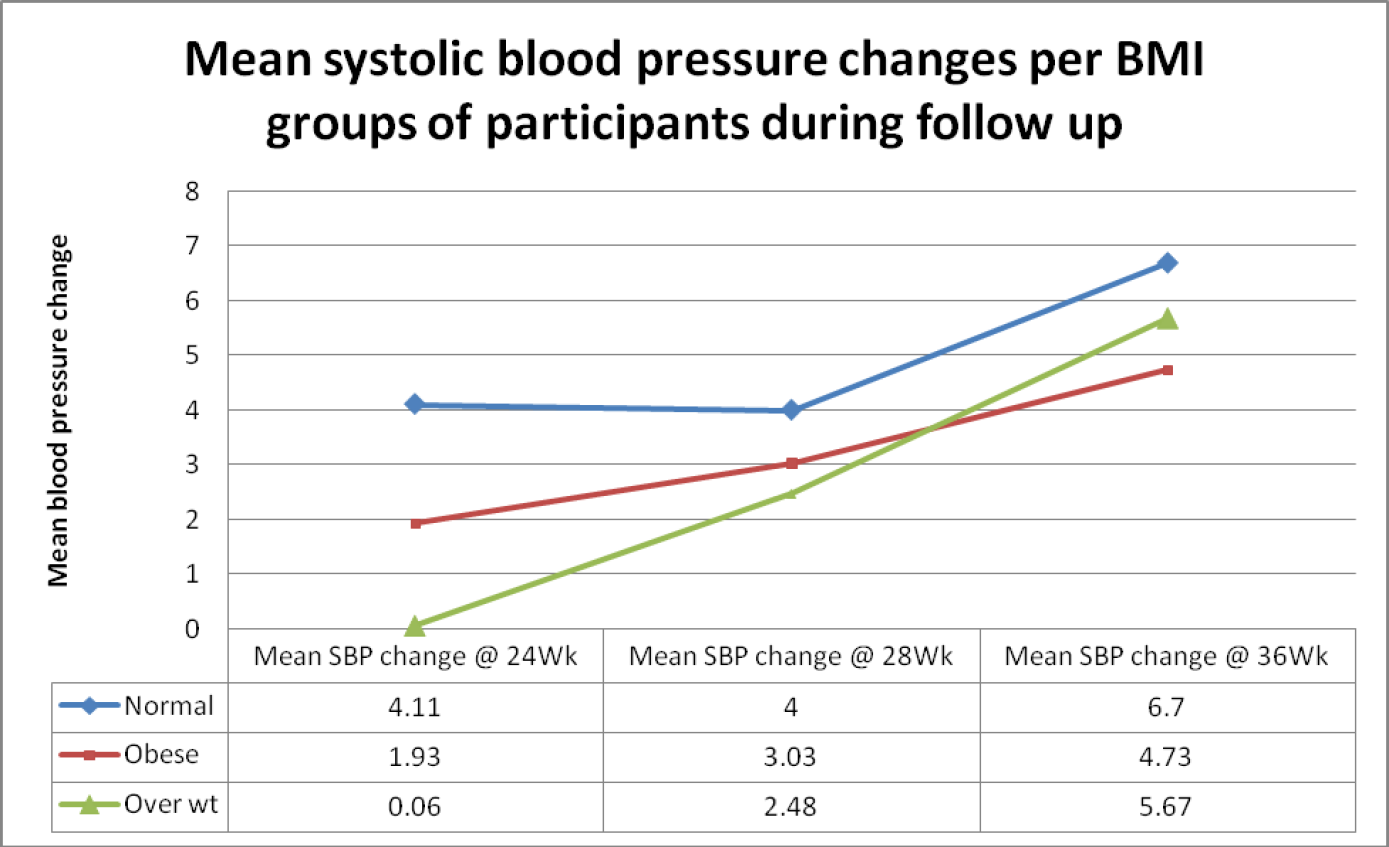
Mean systolic blood pressure changes for BMI groups of participants during follow up.

**Fig 3:**
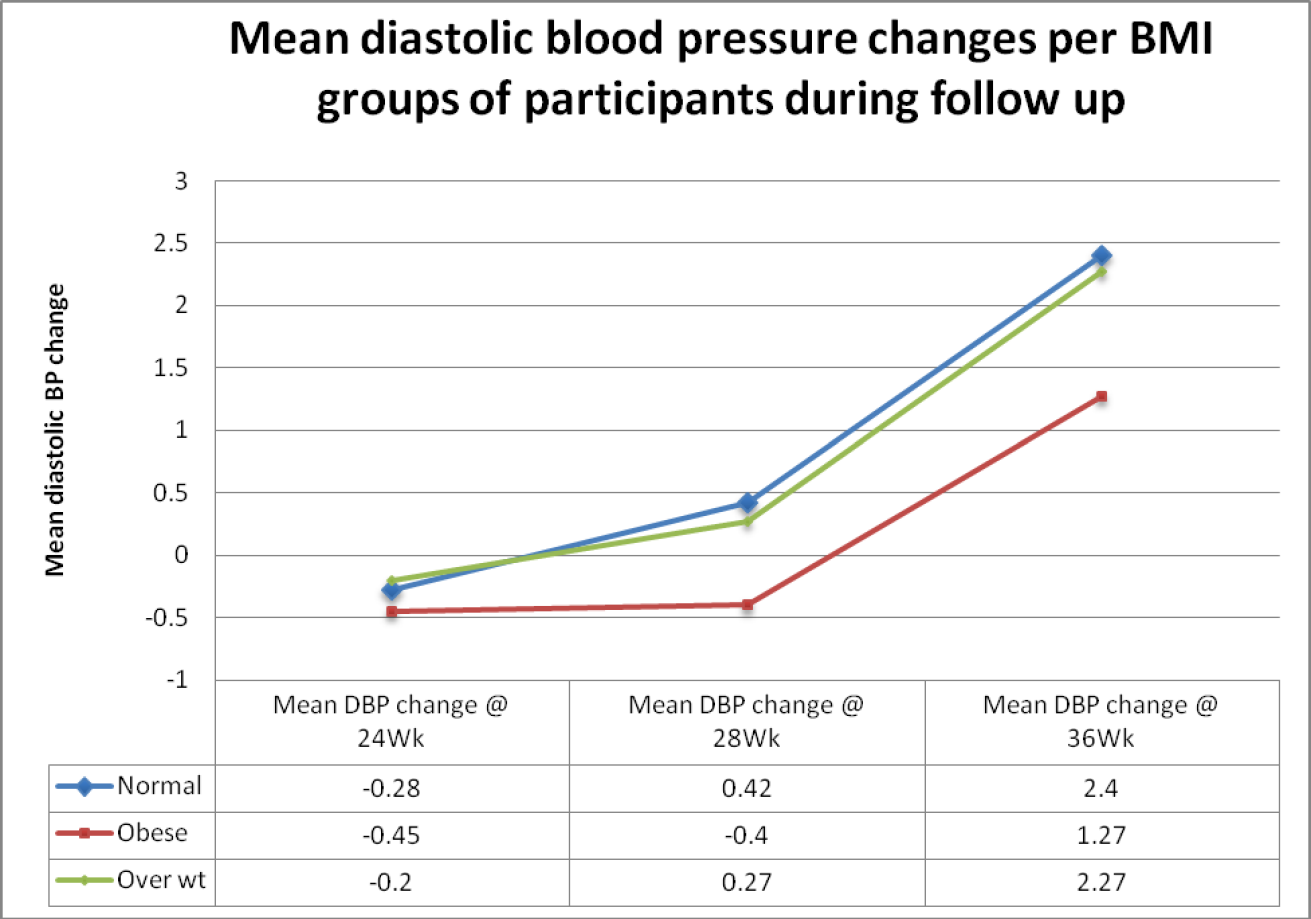
Mean diastolic blood pressure changes for BMI groups of participants during follow up.

Blood pressures (systolic and diastolic) during follow-up at weeks 24, 28 and 36 were compared with baseline blood pressure for participants and the mean changes during follow-up estimated for normal, overweight and obese women. Underweight women were excluded in this analysis because of their small number (3). For women with normal BMI, mean systolic blood pressure changes at the 3 points were 4.11, 4.00 and 6.70 mmHg respectively while mean diastolic blood pressure changes were -0.28, 0.42 and 2.40 mmHg respectively. For obese women however, mean systolic blood pressure changes at the 3 points were 1.93, 3.03 and 4.78 mmHg respectively while mean diastolic blood pressure changes were -0.45, -0.40 and 1.27 mmHg respectively. The differences in the mean blood pressure were however not significant ("Figs 2 and 3"). There were however significant differences in mean pressure changes between those who developed PIH and those who did not at the three points (Table 4).

**Table 4:** Mean blood pressure changes from baseline during follow up among the PIH groups.

Diagnosis of PIH	24 weeks	28 weeks	36 weeks
Systolic blood pressure changes: Mean (SD) mmHg			
All women	1.77 (11.88)	3.13 (12.75)	5.89 (13.66)
No PIH	1.08 (11.74)	2.47 (11.86)	4.73 (12.62)
PIH	8.87 (11.21)	10.47 (17.28)	16.36 (17.89)
p-value	<0.01	<0.01	<0.01
Diastolic blood pressure changes (Mean (SD)mmHg)			
All women	-0.29 (9.51)	-0.39 (10.33)	2.22 (10.37)
No PIH	-0.54 (9.50)	-0.35 (9.87)	1.64 (9.56)
PIH	2.37 (9.35)	6.89 (12.03)	7.47 (15.13)
p-value	0.11	<0.01	<0.01

## Discussion

We found overweight and obese pregnant women in our sample to be younger and multiparous. Other studies have found that women with higher BMI tend to be multiparous, just as we found, but older[23,24]. This could be due to the fact that percentage body fat increases with age[25]. The absence of a significant association between socioeconomic factors such as education and employment and BMI is rather contrary to what other studies have found [26–28] While in the past obese women tended to have lower educational and economic status in higher income countries with the reverse relationship for lower to middle income countries, recent studies show different relationships between obesity and specific socio-economic factors for different populations[29–32].

This study also found significant variations in baseline systolic and diastolic BPs for the three BMI categories, with obese and overweight women having higher BPs compared to women with normal BMI. Several studies have also found that increasing BMI is associated with increasing BPs and increased risk or odds of PIH and its complications[23,24,32]. Most of the studies however used pre-pregnancy weights to estimate BMI, while we used weight at 20 weeks of gestation. It is important that women are educated to know their weights, especially pre-pregnancy. This will be facilitated if pre-pregnancy services are provided by the health system, and also utilized by women before they get pregnant. This will also be an avenue to encourage women to keep a healthy weight or reduce excess weight where necessary before pregnancy, despite recent literature that suggests such gains with respect to reduction in complications of PIH would be minimal[33].

Incidence of PIH amongst our cohort of women was 10.5%, with 44.4% and 33.0% of these women being overweight and obese women respectively. The risk of PIH increased from 24 weeks to 36 weeks of gestation. The PIH incidence of 10.5% was high compared to some other studies[34] but lower compared to that found in other parts of Africa[35,36] and in an Australian nulliparous cohort (12.6%)[26]. Nulliparity has also been associated with increased risk of PIH and this has been a consistent finding as shown in a systematic review[27], but we did not find that in our study. It is not clear to us how the relationship between obesity and multi-parity seen in our study contributes to this finding. Although we excluded women with chronic hypertension at baseline, we found that women with higher baseline BPs still had an increased risk of PIH, which is also in agreement with existing literature[27,33].

During follow up, there were significant differences between the mean BP changes at gestational weeks 24, 28 and 36 compared to baseline, between women who had a diagnosis of PIH and those who did not. There were however no such significant differences between follow-up mean BP changes for the different BMI groups.

A key limitation of this study is the fact that weights used for estimating the BMI were not pre-pregnancy weights. The baseline weights were around 20 weeks and thus some substantial pregnancy weight gain may have taken place, affecting the BMI groups and thus the risk categorization. This limitation occurred mainly because in Ghana most women do not know their pre-pregnancy weights, and no previous studies have estimated how much weight women in this setting are likely to gain by gestational age 20 weeks that can be used in adjusting weights in our analysis. Coupled with this limitation was the fact that we excluded women who were already hypertensive at baseline from the study. Also, follow-up BP measurements were missing for a good number of participants (range 108–132 at the 3 follow-up points) who nevertheless completed the study upon delivery. Most of these participants were in the obese group. This limits our sample for analysis and thus it is possible that there may be underestimation of the risk of PIH amongst our study population. The strength of our study is the fact we used standard BP measurement tool and PIH definition and thus our findings are comparable to studies in other settings.

## Conclusion

The incidence of PIH among our cohort was 10.5% and obese women have a three-fold increased risk of PIH. Pregnant women with systolic BP of greater than 130mmHg after 20 weeks have a high risk of developing PIH. Women should be screened at booking for obesity status. Antenatal protocols should have interventions for prevention or early detection and management of obesity in pregnancy to improve outcomes.

## Supporting information

S1 File (XLS)
